# Safe Management Strategies in Clinical Forensic Autopsies of Confirmed COVID-19 Cases

**DOI:** 10.3390/diagnostics11030457

**Published:** 2021-03-06

**Authors:** Cristoforo Pomara, Monica Salerno, Francesco Sessa, Massimiliano Esposito, Martina Barchitta, Caterina Ledda, Patrizia Grassi, Aldo Liberto, Anna Rita Mattaliano, Venerando Rapisarda, Margherita Ferrante, Antonella Agodi

**Affiliations:** 1 Institute of Legal Medicine, Department of Medical and Surgical Sciences and Advanced Technologies “G.F. Ingrassia”, University of Catania, 95123 Catania, Italy; monica.salerno@unict.it (M.S.); massimiliano.esposito91@gmail.com (M.E.); aldoliberto@gmail.com (A.L.); 2 Department of Clinical and Experimental Medicine, University of Foggia, 71122 Foggia, Italy; francesco.sessa@unifg.it; 3 Department of Medical and Surgical Sciences and Advanced Technologies “G.F. Ingrassia”, University of Catania, 95123 Catania, Italy; mbarchitta@unict.it (M.B.); vrapisarda@unict.it (V.R.); agodia@unict.it (A.A.); 4 Occupazional Medicine, Department of Clinical and Experimental Medicine, University of Catania, 95121 Catania, Italy; cledda@unict.it; 5 Director of Microbiology Section, Analysis Laboratory, San Marco Hospital, 95121 Catania, Italy; patricgrassi@gmail.com; 6 Director of San Marco Hospital, 95121 Catania, Italy; direzionemedicasanmarco@policlinico.unict.it; 7 Environmental and Food Hygiene Laboratory (LIAA), Department of Medical and Surgical Sciences and Advanced Technologies “G.F. Ingrassia”, University of Catania, 95123 Catania, Italy; marfer@unict.it

**Keywords:** COVID-19, autopsy safe management, clinical forensic autopsy, disinfection, post-mortem swab

## Abstract

To date, there is poor evidence on the transmission of infection in individuals handling the bodies of deceased persons infected with SARS-CoV-2 and in particular, during autopsies. The aim of this study was to demonstrate that when appropriate strategies are adopted autopsy is a safe procedure with a minimal infection risk for all subjects involved (pathologists, technical personnel, and others) when proper strategies are adopted. We performed 16 autopsies on cadavers of persons who had died with confirmed COVID-19 with different post-mortem intervals (PMI). To confirm the presence of SARS-CoV-2 RNA, for each autopsy, 2 swabs were sampled from lungs, while to evaluate environmental contamination, 11 swabs were taken at three different times: T0 (before autopsy), T1 (at the end of the autopsy, without removing the corpse), and T2 (after cleaning and disinfecting the autopsy room). Specifically, 2 swabs were sampled on face shields used by each pathologist, and 4 swabs were collected on the autopsy table; 4 swabs were also collected from walls and 1 from floor. Lung swabs confirmed the presence of SARS-CoV-2 RNA in all cases. Environmental swabs, collected at T0 and T2 were all negative, while swabs sampled at T1 were shown to be positive. Interestingly, no association was shown between PMI length and environmental contamination. Infection control strategies for safe management of clinical forensic autopsies of bodies with suspected or confirmed COVID-19 are also described.

## 1. Introduction

The Severe Acute Respiratory Syndrome Coronavirus-2 (SARS-CoV-2) pandemic is associated with a broad spectrum of clinical manifestations, mainly characterized by an acute respiratory syndrome associated with a multi-organ syndrome, causing more than 1.9 million deaths worldwide (based on the data reported by the World Health Organization (WHO). As of 10 January 2021, there have been 1,914,378 deaths from 88,120,981 confirmed cases) [1]. To date, the pathophysiological mechanisms causing death and the therapeutic strategies to counteract them remain elusive. Although a significant contribution has come from autopsy studies, cadaveric studies are still limited despite the fact that autopsies represent the gold standard to identify the cause of death. Autopsy is also a valuable tool to obtain accurate mortality statistics, which could become essential for public health and health service planning [2]. Although autopsy should be considered a safe procedure, the autopsy rate is constantly being reduced, to the point that it could be considered extinct or on the way to extinction as recently argued by De Cock et al. in *The New England Journal of Medicine* [3]. One of the reasons for the poor use of autopsy is undoubtedly linked to the uncertainty of safety when handling the corpses of patients who died following COVID-19 [4,5]. In this scenario, especially in the first phase of the outbreak of COVID-19, the so-called “lockdown of the science” occurred, considering that a very low number of autopsies had been performed [6]. Nevertheless, in a background of limited scientific knowledge and limited evidence of the COVID-19 outbreak, autopsy has been a crucial tool to better understand the pathological features of COVID-19, clarifying SARS-CoV-2 pathophysiology, as well as improving prevention and treatment [7,8,9,10]. 

In March 2020, the WHO published an interim guidance titled “Infection prevention and control for the safe management of a dead body in the context of COVID-19”, suggesting that there was no evidence of persons having become infected from exposure to the bodies of a subject who died from/with COVID-19, recommending several precautions that the pathologist should apply during post-mortem investigation [11]. Centers for Disease Control and Prevention (CDC) also published a post-mortem guidance, frequently reviewed during the pandemic [12]. Although the great efforts of the scientific community to improve the knowledge about post-mortem investigations, to date, several important concerns have not been clarified to encourage the continued use of autopsies.

The aim of this study was to demonstrate that when appropriate strategies during post-mortem investigation concerning handling the bodies of deceased persons infected with COVID-19 are adopted, autopsy is a safe procedure with a minimal infection risk for all involved subjects (pathologists, technical personnel, and others).

## 2. Materials and Methods

### 2.1. Samples

A total of 16 complete autopsies (8 males and 8 females) were performed on subjects who died due to confirmed SARS-CoV-2 infection. Age was in the range of 50 to 93 years (mean 75.1 +/− 14.8 SD, median 76.5 years). Before death, all nasopharyngeal swabs collected from the subjects enrolled in the present study tested positive at the COVID-19 reverse transcription polymerase chain reaction (rRT-PCR assay). The main cause of death was interstitial pneumonia with fibrosis that involved five subjects (cadaver ID 1,6,8,10,14); moreover, four patients died from pulmonary edema (cadaver ID 2,4,5,12), three patients died from Multiple Organ Dysfunction Syndrome (MODS) (cadaver ID 13, 15,16), two from septic shock (cadaver ID 7,11), patient #9 died from cardiac failure, and patient #3 died from Acute respiratory distress syndrome (ARDS). The post-mortem interval (PMI) ranged from 1 day to 78 days (mean 27 +/− 30.5 SD, median 3 days). Before autopsy, both molecular and antigen tests were performed on all personnel involved in the post-mortem procedures obtaining negative results. Moreover, during the study period, they were constantly monitored (as programmed by our University, every 5 days) resulting negative at all steps. All procedures performed in the study were approved by the Scientific Committee of the Department of Medical and Surgical Sciences and Advanced Technologies “G.F. Ingrassia”, University of Catania, (record n. 21/2020) and were performed in accordance with the 1964 Helsinki Declaration and its later amendments or comparable ethical standards. The study was conducted according to the Italian Law n° 81/2008 concerning the safety of workers and workplace of public Hospitals. The Director of San Marco Hospital authorized the use of anonymous data according to Italian law. No informed consent is required to use information from deceased persons where the same information is strictly indispensable and relevant for scientific and research purposes. Finally, since the proposed experimental model refers to the assessment of environmental contamination during autopsy procedures, no sensitive patient data are treated.

### 2.2. Autopsy Room (A.R.)

According to international guidelines [9,11,13], autopsies were conducted in Airborne Infection Isolation Rooms (AIIRs) with a negative pressure of 9.7 air changes per hour (ACH). Doors were kept closed during the procedure, and the AIIR room air was exhausted directly outdoors, away from windows, areas of human traffic or gathering spaces.

The AR was 57 m^2^, 2.7 m high, and 153.9 m^3^ volume. The delivery airflow rate was 1136 m^3^/h and the return 1850 m^3^/h. The AR temperature was 21.8 °C. The filtration system consisted of:

Filter MULTICEL mod. 3mc14 305 × 610 × 292
Type—HEPA 20 R01
Integral efficiency (%)—99.995 at MPPS
Local efficiency (%)—99.975 AT MPPS
Class to en 1822—H 14
Size (mm)—305 × 610 × 292
Airflow (m^3^/h)—1700
Resistance to airflow (Pa)—250
Reference number—20 13
Serial number—600777.

Wall 1 is located near the forensic staff exit door. Wall 2 is located near the steel cabinet. Wall 3 is located near the forensic staff entrance door and the corpse entrance door (hermetically sealed door). Wall 4 is located near the sink.

The personal protective equipment (PPE) used were double surgical gloves interposed with a layer of cut-proof synthetic mesh gloves, fluid-resistant or impermeable isolation gown, waterproof apron, goggles or face shield, national institute for occupational safety and health (NIOSH) -approved disposable N-95, air-purifying respirators (PAPRs) with HEPA filters, surgical scrubs, shoe covers, and surgical cap. After the autopsy, PPE were thrown in the appropriate waste receptacle. Reusable PPE (like face shields) were disinfected accordingly before reuse.

The use of a low-pressure water jet to avoid vaporization was limited when it was necessary only to a few, cleaning procedures of the instruments.

### 2.3. Autopsy Protocol

To assess the efficacy of the autopsy safety procedure in deceased COVID-19 positive subjects, and the effectiveness of the disinfection procedure, a standardized pre- and post-disinfection swab collection procedure was performed.

The tool used for swab collection was in accordance with the CDC guidelines [13].

The standardized procedure was divided into three stages:

T0—before the autopsy;
T1—at the end of autopsy (without removing the corpse);
T2—after the autopsy (after cleaning and disinfection of the AR).

#### 2.3.1. T0—Before the Autopsy

**Figure 1:**
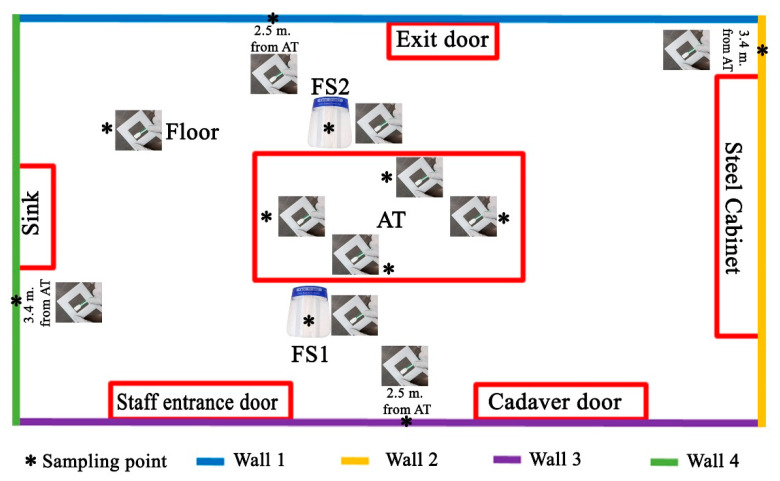
The scheme of environmental sampling of the autopsy room (A.R.): as indicated in the main text, for each autopsy 11 swabs were sampled.

The cadaver was outside the AR. The following environmental swabs were collected, before each autopsy (Figure 1).

Autopsy table (AT) (4 swabs in total): (1) 40 cm from the head on a surface of 6 cm^2^; (2) 40 cm from the right arm on an area of 6 cm^2^; (3) 40 cm from the left arm on an area of 6 cm^2^; and (4) 40 cm from the feet on an area of 6 cm^2^;

Face shield (FS) (2 swabs in total) of the forensic pathologists performing the section of the cadaver on a surface of 6 cm^2^. One swab was collected on the FS of the forensic pathologist placed to the right side of the cadaver (FS1) and one swab (FS2) was collected on the FS of the forensic pathologist to the left side of the cadaver;

Four walls of the autopsy room (A.R.), at a height of 1.3 m and on an area of 6 cm^2^ (4 swabs in total);

Floor of the AR, at a distance of 1.5 m from the autopsy table (head-right side angle, n.1 swab in total).

As summarized in Figure 1, at the end of the operation, we collected 11 swab samples for each of the 16 autopsies (total swabs 176).

#### 2.3.2. T1—At the End of Autopsy

**Figure 2:**
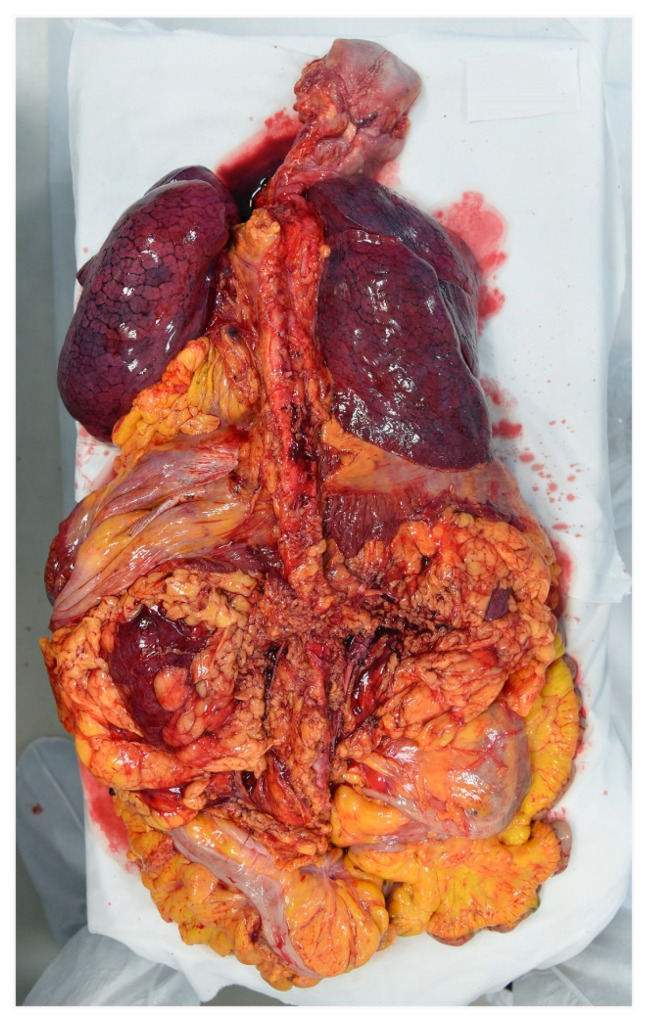
Anterior face of lung on the viscera obtained through the Letulle technique.

The cadaver was placed on the autopsy table. All 16 autopsies of COVID-19 cases were conducted according to the Letulle technique, removing all the viscera in order to reduce environmental contamination [14]. Indeed, an extraction of the oral (tongue, oropharynx, hypopharynx), cervical (larynx, trachea) and thoracic (tracheal bifurcation, major bronchi, lungs) respiratory structures, as well as abdominal structures (kidney, gastrointestinal system) was carried out, with final resection from the surrounding structures (iliac arteries and vein) (Figure 2). A handsaw with a chain-mail glove was used to saw the skull. The brain was preserved either in its entirety or, in cases of decomposed bodies, a part was removed and placed in a container. All organs were fixed in formalin.

**Figure 3:**
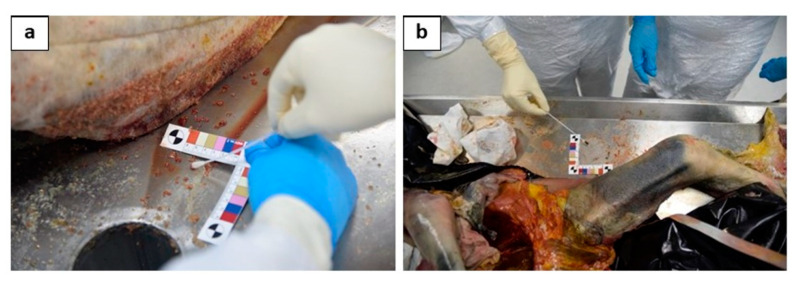
The particular of the sampling of the 2 swabs performed on the autopsy table: one performed 40 cm from the head on a surface of 6 cm^2^, near to the right upper arm (**a**); (2) another one performed 40 cm from the left arm on an area of 6 cm^2^, near to the left femur(**b**).

Before the fixation procedures, 2 swabs were taken from the lower respiratory tract (primary bronchi): the first to the right bronchus, the second to the left bronchus. Before the cleaning and disinfection operation, 11 environmental samples were also collected at this time (T1). As indicated in Figure 1, the sampling has been performed as described:

AT (4 swabs in total): (1) 40 cm from the head on a surface of 6 cm^2^ (Figure 3a); (2) 40 cm from the right arm on an area of 6 cm^2^ (Figure 3b); (3) 40 cm from the left arm on an area of 6 cm^2^; and (4) 40 cm from the feet on an area of 6 cm^2^.

FS (2 swabs in total) of forensic pathologists who made the section of the cadaver on a surface of 6 cm^2^. FS1 was the face shield of the forensic pathologist to the right side of the cadaver. FS2 was the face shield of the forensic pathologist to the left side of the cadaver;

Four walls of the autopsy room, at a height of 1.3m and on an area of 6 cm^2^ (4 swabs in total);

Floor of the AR, at a distance of 1.5 m from the AT (head-right side angle, 1 swab in total).

At the end of the operation we collected 11 swab samples for each of the 16 autopsies (176 total swabs).

#### 2.3.3. T2—Disinfection Procedures after the Autopsy

The cadaver was outside the autopsy room. According to guidelines [11,13] the disinfection procedure was performed with a minimum concentration of 0.1% (1000 ppm) sodium hypochlorite (bleach). Moreover, the complete disinfection of the personnel involved in the autopsy procedures was performed before leaving the room at the end of the autopsy through a nebulization procedure of all PPE products (such as overalls, gloves, face shield, etc.).

After disinfection, 11 environmental swabs were also collected at time T2. Particularly, all swabs were taken following the described procedures:

AT (4 swabs in total)—(1) 40 cm from the head on a surface of 6 cm^2^;—(2) 40 cm from the right arm on an area of 6 cm^2^; (3) 40 cm from the left arm on an area of 6 cm^2^; (4) 40 cm from the feet on an area of 6 cm^2^.

**Figure 4:**
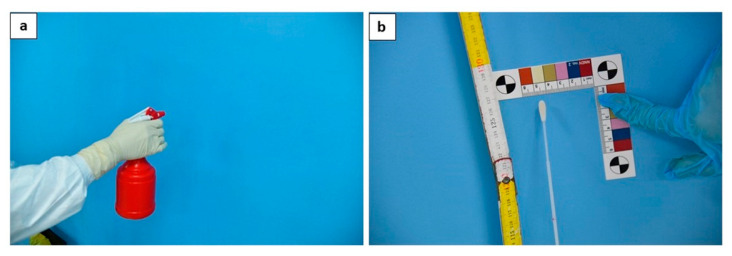
The disinfection procedure is performed through nebulization (**a**); at the end of the procedures, one swab for each wall was taken (**b**).

FS (2 swabs in total) of forensic pathologists who made the section of the cadaver on a surface of 6 cm^2^; F.S.1 was the face shield of the forensic pathologist to the right side of the cadaver. F.S.2 was the face shield of the forensic pathologist to the left side of the cadaver;

Four walls of the autopsy room (Figure 4), at a height of 1.3 m and on an area of 6 cm^2^ (4 swabs in total);

Floor of the AR, at a distance of 1.5 m from the autopsy table (head-right side angle, 1 swab in total).

### 2.4. Swab Analysis

Each container was labelled with the identification code to identify the subject, the sample, and the date of collection. The procedure was as follows: (i) insertion of sterile tubes containing the swabs into a secondary container; (ii) placement of the containers inside a sealable clean plastic bag; (iii) if possible, insertion of the sealable bag in an additional container for biological samples; (iv) transfer outside the autopsy area and delivery to an operator equipped with disposable nitrile gloves for transport. Then the swabs were sent to the San Marco Hospital of Catania, where real-time reverse transcriptase-polymerase chain reaction (rRT-PCR) testing was performed. Afterwards, swabs were taken to and accepted by the microbiology laboratory in a maximum time of 30 min. Swab samples placed in viral transport medium were analyzed by rRT-PCR for SARS-CoV-2 RNA detection. All the swabs collected during the first 9 autopsies (cadaver ID 1-9, Table 1) were analyzed using the Aptima SARS-CoV-2 Assay (Hologic, Inc., San Diego, CA, USA), according to the manufacturer’s instruction, with automatic data system analysis software (Panther Fusion, Hologic, Marlborough, MA, USA) for identifying positive samples. The other collected swabs (cadaver ID 10-16) were analyzed by multiplex rRT-PCR assay using GeneXpert Xpress SARS-CoV-2 on the CFX96 real-time (Cepheid, Sunnyvale, CA, USA). Both assays are designed to investigate genes specific for SARS-CoV-2 according to the US and Chinese Centers for Disease Control [15]; moreover, a recent study demonstrate an overall agreement of 99% between the Cepheid Xpert Xpress SARS-CoV-2 assay and the GeneXpert Xpress SARS-CoV-2 assay confirming their position as robust and comparable diagnostic options for the identification of SARS-CoV-2 [16]. All the procedures to prevent specimen contamination and PCR carryover were rigorously respected at all phases.

### 2.5. Statistical Analysis

To perform the statistical analysis, the rate of positive swabs was calculated for each autopsy and for each sampled area: this value is obtained dividing the number of environmental positive swabs by the total number of environmental collected swabs. Considering that the swabs collected at T0 and at T2 were all negative, the analysis was performed only for the swabs collected at T1. Furthermore, all environmental swabs collected at T1 were split into two subgroups following the PMI criterion: the group named “short PMI” (where “PMI” indicates the interval time elapsed from death to the autopsy ranging between 1 and 5 days) was composed of the swabs collected during the autopsy performed on a corpse with a PMI ≤ 5 days. It is important to note that usually a PMI of 5 days could elapse between death and the post-mortem investigation both in clinical and forensic autopsies. The group named “long PMI” was composed of data obtained from the swabs collected during post-mortem investigation performed on subjects with a PMI ranging from 53 to 78 days.

Moreover, the data were divided following the “area of sampling” criterion: the swabs collected on the surfaces located near the corpse (swabs performed on FS and AT) made up the group named “Near swabs” (6 swab samples for each autopsy), while the swabs collected on the walls and on the floor made up the group named “Far swabs” (5 swab samples for each autopsy).

Data were analysed with the software SPSS 22.0 package for Windows. Independent *t*-test for two samples was used to determine any statistically significant differences in positive rates between subgroups (“short PMI” vs. “long PMI” and “near swabs” vs. “far swabs”).

## 3. Results

### 3.1. Result of Low Respiratory Swabs

The molecular test for SARS-CoV-2-RNA using quantitative rRT-PCR was performed on all samples of low respiratory airways collected at time T1 (at the end of autopsy). All 32 swabs (16 on the right bronchus and 16 on the left bronchus) were positive. The positive results were not influenced by the period of time (ranging from 5 to 54 days) elapsed between the ante-mortem COVID-19 diagnostic test and the post-mortem swabs (COVID-19 rRT-PCR assay performed on post-mortem swab). The crucial aspect is the persistence of the RNA virus in all decomposed bodies up to a PMI of 78 days (Table 1).

### 3.2. Result of Environmental Swab

At time T0 (before the autopsy), all environmental swabs collected from the AR (total environmental swabs 176) were negative for the RNA of SARS-CoV-2 as summarized in Supplementary Table S1.

At time T1 (at the end of autopsy), the environmental swabs of the AT showed the following results: the rate of positive swabs (presence of RNA of SARS-CoV-2) of the AT on the right was 68.75% (*n* = 11/16); the rate of positive swabs of the AT on the left was 81.25% (*n* = 13/16); the rate of positive swabs of the A.T. head was 62.5% (*n* = 10/16); the rate of positive swabs of the foot of the AT was 68.75% (*n* = 11/16).

The FS swabs of the two forensic pathologists who performed the autopsy gave a positive rate of 15.6% (*n* = 5/32). Nevertheless, the positive results did not occur during the same autopsies. FS1 displayed positivity to the swab collected during autopsies 2 and 4 (*n* = 2/16), while FS2 displayed positivity to the swab during autopsies 1, 12, and 13 (*n* = 3/16).

The environmental swabs of the autopsy room wall (AW) (64 total swabs; total positive rate 4/64, 6.25%) showed the following results: AW1 was 0% (*n* = 0); the rate of positive swabs of AW2 was 6.25% (*n* = 1/16); the rate of positive swabs of AW3 was 12.5% (*n* = 2/16); the rate of positive swabs of AW4 was 6.25% (*n* = 1/16).

The rate of positive swabs of the floor of the A.R. was 37.5% (*n* = 6/16).

All environmental swab results at time T1 are summarized in Table S2.

All environmental swabs collected at T1 were split into two groups following the PMI criterion: these are the group named “short PMI” and the group named “long PMI”. The results are summarized in Table 2.

As summarized in Table 2, to perform the statistical analysis, the rate of positive swabs was calculated for each autopsy (mean values of positive “short PMI” = 0.29 ± 0.22; mean values of positive “long PMI” = 0.40 ± 0.18). Applying the independent *t*-test, no statistically significant differences were detected (*p* > 0.05): this result demonstrated that environmental contamination is not related to the fact that the corpse is in an advanced state of decomposition.

Moreover, the data were divided under the “area of sampling” criterion: the swabs collected on the surfaces located near the corpse made up the group named “Near swabs”, while the swabs collected on the wall and on the floor made up the group named “Far swabs” (Table 3). A statistically significant difference was reported (*p* < 0.05) comparing the rate of total near positive swabs (mean values 0.52 ± 0.29) versus the rate of total far positive swabs (mean values 0.12 ± 0.16).

Applying the *t*-test, no statistically significant differences were detected in the two main groups (“long PMI” vs. “short PMI”) both comparing the results of “Near swabs” subgroup (*p* < 0.05) and the results of “Far swabs” subgroup (*p* < 0.05).

Finally, evaluating the intra group variability of the “long PMI” group (“Near swabs” sub-group vs. “Far swabs” sub-group) the rate of positive “Near swabs” was significantly higher than the rate of positive “Far swabs” (*p* < 0.05). The same result was obtained in the “short PMI” subgroup: the rate of positive “Near swabs” was significantly higher than the rate of positive “Far swabs” (*p* < 0.05). All statistical test values are summarized in Table 4.

Finally, at time T2, after the disinfection procedure and when the cadaver was outside the autopsy room, all environmental swabs were negative for SARS-CoV-2.

All environmental swab results at time T2 are summarized in Table S3.

## 4. Discussion

Although a low risk of SARS-CoV-2 transmission by different surfaces in real-life conditions has been reported, the persistence of coronaviruses including SARS-CoV-2 on inanimate surfaces for days has been demonstrated [17,18]. To the best of our knowledge, no study has been performed to evaluate the safe management of bodies of deceased persons with suspected or confirmed COVID-19 [19].

Edler et al. [20], performed 30 autopsies where they diagnosed SARS-CoV-2 infection through swabs, followed by PCR post-mortem, of the lung tissue at the time of dissection. The maximum PMI was 12 days. The sensitivity of the positivity of swab decreased with a longer PMI [13]. As remarked in a very recent review [21], there is poor scientific knowledge about the best practice for handling a COVID-19 positive corpse, although the scientific community has proposed several guidelines and recommendations.

PPE are required when performing an autopsy in suspected COVID-19 subjects [22]. The necessary PPE are: disposable gloves, FFP3 respiratory filters, goggles or protective visor, disposable long sleeved gown or waterproof suit, and disposable overshoes [23,24]. Because of the risk of puncture, cut-resistant gloves should be used. Used PPE should be appropriately disposed of in suitable containers. It is necessary to avoid contact with the face and mouth, and to wash hands after the autopsy [25,26].

In the present study, 16 complete autopsies were performed on subjects all ante-mortem positive for SARS-CoV-2. A standardized swab collection procedure was performed, and was divided into three stages: T0—before the autopsy, when the body was not in the AR; T1—at the end of the autopsy, before removing the corpse; T2—after the autopsy, without the presence of the corpse in the AR and after carrying out the disinfection procedure. At time T0 (before the autopsy), when the cadaver was outside the AR, all environmental swabs were negative for SARS-CoV-2. Unlike previous studies [27,28], in the present study, at time T1 (at the end of the autopsy), all lower respiratory tract swabs (right bronchus and left bronchus) were positive. However, because a viral culture was not performed, we are unable to confirm the presence of a viable virus.

As discussed in the Section 3, no statistically significant difference was reported between the two tested groups (“long PMI” vs. “short PMI”).

Although it could be supposed that a long PMI reduced environmental contamination, our data demonstrated that it is not true.

The FS swabs of the two forensic pathologists who performed the autopsy gave a positive rate of 31%. The positive swabs of the FS were due to body fluid splatters during the autopsy. However, the distribution was not the same. FS1 displayed positivity to the swab during the autopsies 2, 4. FS2 displayed positivity during autopsies 1, 12, 13. Therefore FS never gave a positive result to swabs during the same autopsy. The different positivity of FS of the two forensic pathologists depended on who performed some operations such as head dissection, airway sampling or airway opening.

Our results confirmed that the area near the corpse is the most contaminated surface. Despite the fact that we were unable to confirm the presence of a viable virus on the environmental surfaces sampled, these findings suggest that pathologists should pay attention during the autopsy, particularly in the area near the body, practicing all suggested countermeasures to reduce room contamination. Notably, this study shows the importance to plan different autopsy areas (such as “clean area”, “transition area”, “dirty area”), ensuring an adequate physical space related to the number of personnel involved in the autopsy.

The virus normally survives for a few hours outside the host, but this may extend to days in some conditions [17]. It is essential to neutralize the virus by the use of bleach and ethanol solutions. According to van Doremalen et al. [18], the virus survives 3 h post aerosolisation, up to 4 h on metal, more than one day on paper/board and up to 2–3 days on plastic and stainless steel. Furthermore, disinfection is required for any non-disposable equipment being used during the autopsy. The decontamination procedure is crucial, and all the objectives are to ensure the health and safety of those carrying out the handling of the corpse and prevent the unnecessary spread of contamination. The decontamination and disinfection approach should be considered part of the autopsy to ensure that the most effective method is adopted. Upon completion of the autopsy, before removing any equipment from the temporary holding area, care must be taken to assure that it does not present a cross-contamination hazard [25]. Yaacoub, S. [19] affirmed that it is recommended to perform regular disinfection using hospital disinfectants or with 0.1% sodium hypochlorite (dilution 1:50 if household bleach at an initial concentration of 5% is used) or a neutral detergent, followed by a 70% concentration of ethanol.

In the present study, an environmental disinfection procedure was performed according to previous guidelines [11,13] with a minimum concentration of 0.1% (1000 ppm) sodium hypochlorite (bleach); moreover, complete disinfection of the personnel involved in the autopsy procedures was performed before leaving the room at the end of the autopsy through a nebulization procedure of all PPE products (such as overalls, gloves, face shield, etc.). and all environmental swabs were negative for SARS-CoV-2 (100% negative, 0% positive).

A thorough knowledge of the eventual persistence of pathogens in deaths related to infectious diseases is crucial to secure an approach to complete autopsy performance in which the operators can be fully aware of the potential high biological risks before exposure [29]. An effective way post-mortem staff can effectively reduce the risks associated with necropsies is the awareness of the bodies’ infective status. A noteworthy result of this study is to ensure an accurate disinfection procedure for personnel who perform autopsies with high biological risk. Safety of our disinfection procedure is guaranteed through the negativity of the post-disinfection swabs of the entire environment and of all objects.

Moreover, the results of the present study support the effectiveness of adherence to international guidelines and/or recommendations during the post-mortem investigation on infection control among the pathology staff (technicians, biologists, pathologists). For these reasons, we disagree with the commentary of Sapino et al. [30], where the authors reported that autopsies should be restricted to well-motivated cases. On the contrary, we stress the importance of autopsies, especially in the management of unknown diseases.

After the autopsy, the staff involved in performing these complete autopsies underwent nasopharyngeal swabs for SARS-CoV-2 and were negative (the autopsies involved an exposure time for the medical and technical staff of 2 h).

It is important to note that this study was conducted on COVID-19 corpses during autopsy. This represents the strength of this study: indeed, the data obtained allowed us to evaluate environmental contamination during a COVID-19 autopsy.

The main limitation of this study is related to the fact that we detected the positivity of swab samples collected during autopsy procedures through real-time PCR; without assessing the risk of causing an infection. This limitation is reported in several previous studies: for this reason, it is fundamental to remark that before sending alarming messages, the forensic community is called on to fully comprehend the weight of the evidence.

## 5. Conclusions

**Figure 5:**
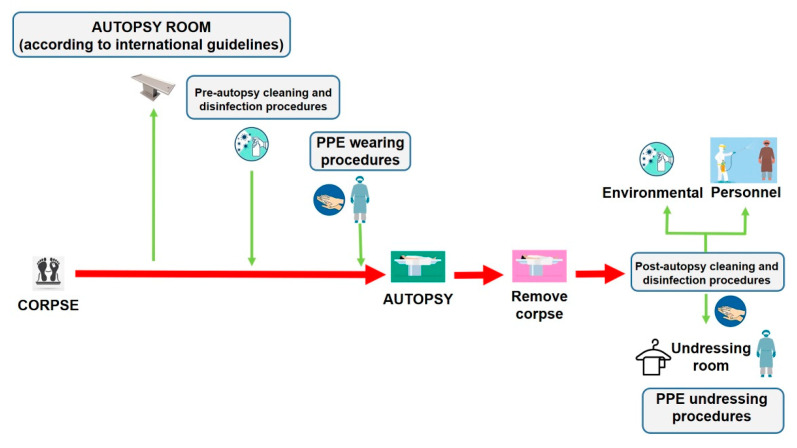
The management of corpses of persons with suspected or confirmed COVID-19 to perform autopsy, should be made following all disinfection procedures described in the current paper. In particular, after removing the corpse, it is necessary to adopt cleaning and disinfection procedures both in the autopsy room and on the personnel involved in the post-mortem investigation before leaving the room, through a nebulization procedure of all PPE products (the personnel is depicted in purple). In this manner, when the personnel (in this case depicted in blue) arrive in the changing area, they perform hand hygiene with specific products, and start the changing procedures in a safe manner.

Autopsies provide crucial public health information, particularly during a pandemic infection such as COVID-19. As a scientific community, we are called on to face this global threat and to defeat it with all the available tools necessary. Despite the improvement and reinforcement of any study method in every field of medicine and science, encouraging the autopsy practice as a tool of investigation could also, help physicians to define an effective treatment to reduce mortality [6]. Reviving the autopsy practice can provide useful information to be matched with clinical data to obtain a better knowledge of the pathogenesis of this novel coronavirus disease [3,31,32,33]. We have summarized the main procedures that should be respected to minimize the infection risks for the personnel involved in post-mortem investigations of persons who died with suspected or confirmed COVID-19 (Figure 5).

Additional studies on the potential transmission of COVID-19 from dead bodies, or samples of them, to individuals and contextual factors are required to provide further evidence, however, to date, the present study is the first that has demonstrated that, with appropriate precautions, there is limited risk to autopsy personnel. In light of these results the autopsy of bodies with confirmed COVID-19 should be considered a safe procedure and as such its practice should be encouraged throughout the scientific community.

## Figures and Tables

**Table 1:** Molecular test for SARS-CoV-2 using quantitative rRT-PCR performed at time T1 (during the autopsy) is positive in all samples of low respiratory airways. These results are neither influenced by the CDTPMS (period of time elapsed between the COVID-19 diagnostic test and the post-mortem swabs) nor by PMI.

SARS-CoV-2 RNA Test Results from Corpses						
Cadaver ID	Age	Sex	COVID-19 rRT-PCR Assay Diagnostic Test Performed on Post-Mortem Swab (Days)	PMI (Days)	Molecular Test for SARS-CoV-2 Using Quantitative RT-PCR (Right Bronchus)	Molecular Test for SARS-CoV-2 Using Quantitative RT-PCR (Left Bronchus)
1	50	F	58	54	Pos	Pos
2	91	F	NA	58	Pos	Pos
3	92	M	54	54	Pos	Pos
4	83	M	NA	61	Pos	Pos
5	89	M	NA	64	Pos	Pos
6	88	F	54	50	Pos	Pos
7	93	F	NA	78	Pos	Pos
8	79	F	19	1	Pos	Pos
9	72	F	5	5	Pos	Pos
10	86	F	9	1	Pos	Pos
11	64	M	30	1	Pos	Pos
12	74	F	18	1	Pos	Pos
13	59	M	9	1	Pos	Pos
14	59	M	7	1	Pos	Pos
15	51	M	17	1	Pos	Pos
16	72	M	5	1	Pos	Pos

Abbreviations: NA, not available; CDTPMS, COVID-19 Diagnostic Test Post-Mortem Swab; PMI, Post-Mortem Interval; Pos, positive result.

**Table 2:** All environmental swabs collected at T1 were split into two groups: “Short PMI” and “Long PMI”. The statistical analysis was performed obtaining the positive rate (ratio among positive environmental swabs and total sampled swabs).

Comparison of Positive Rates between Short PMI and Long PMI					
Short PMI			Long PMI		
Cadaver ID	Positive Swabs/Total Swabs (Percentage)	Rate (Positive Environmental Swabs/Total Swabs)	Cadaver ID	Positive Swabs/Total Swabs (Percentage)	Rate (Positive Environmental Swabs/Total Swabs)
1	7/11 (63.6%)	0.63	8	1/11 (9%)	0.09
2	6/11 (54.5%)	0.54	9	5/11 (45.4%)	0.45
3	5/11 (45.4%)	0.45	10	3/11 (27.2%)	0.27
4	5/11 (45.4%)	0.45	11	1/11 (9%)	0.09
5	4/11 (36.3%)	0.36	12	6/11 (54.5%)	0.54
6	3/11 (27.2%)	0.27	13	7/11 (63.6%)	0.63
7	1/11 (9%)	0.09	14	0 (0%)	0
			15	2/11 (18.1%)	0.18
			16	4/11 (36.3%)	0.36

Abbreviations: PMI, Post-Mortem Interval.

**Table 3:** All environmental swabs collected at T1 were split into two groups: “Short PMI” and “Long PMI”. Moreover, the same groups were divided into two subgroups: Near swabs (all swabs collected on the FS and on the AT) and far swabs (all swabs collected on the walls and on the floor).

Comparison of Positive Rates between “Near Swabs” and “Far Swabs” by Long PMI and Short PMI					
Long PMI			Short PMI		
Cadaver ID	NEAR SWABS (Autopsy Table + Face Shield)	FAR SWABS (Wall + Floor)	Cadaver ID	NEAR SWABS (Autopsy Table + Face Shield)	FAR SWABS (Wall + Floor)
	Rate (Positive Environmental Swabs/Total Swabs)	Rate (Positive Environmental Swabs/Total Swabs)		Rate (Positive Environmental Swabs/Total Swabs)	Rate (Positive Environmental Swabs/Total Swabs)
1	0.83	0.4	8	0.16	0
2	0.83	0.2	9	0.66	0.2
3	0.66	0.2	10	0.5	0
4	0.83	0	11	0.16	0
5	0.66	0	12	0.83	0.2
6	0.5	0	13	0.83	0.4
7	0.16	0	14	0	0
			15	0.33	0
			16	0.33	0.4
**Mean values**	0.64 ± 0.24	0.11 ± 0.15	**Mean values**	0.42 ± 0.3	0.13 ± 0.17

**Table 4:** Results of *t*-test for each statistical analysis performed.

Results of *t*-Test for Each Statistical Analysis Performed					
	Positive Rate of Total near Swabs vs. Positive Rate of Total Far Swabs	Positive Rate of near Swabs Long PMI vs. Positive Rate of Far Swabs Long PMI	Positive Rate of near Swabs Short PMI vs. Positive Rate of Far Swabs Short PMI	Positive Rate of near Swabs Long PMI vs. Positive Rate of near Swabs Short PMI	Positive Rate of Far Swabs Long PMI vs. Positive Rate of Far Swabs Short PMI
***t*-test values**	*p* < 0.05	*p* < 0.05	*p* < 0.05	n.s.	n.s.

Abbreviations: n.s., not significant.

## Data Availability

The data presented in this study are available on request from the corresponding author. The data are not publicly available due to privacy restrictions.

## Supplementary Materials

The following are available online at https://www.mdpi.com/2075-4418/11/3/457/s1, Table S1: Analysis of swab sampling at time T0 (before autopsy). Table S2: Analysis of swab sampling at time T1. Table S3: Analysis of swab sampling at time T2.
