# Supplementary material for: Safe Management Strategies in Clinical Forensic Autopsies of Confirmed COVID-19 Cases
Source: Diagnostics (Basel). 2021 Mar 6;11(3):457. doi: 10.3390/diagnostics11030457 (PMC7999752; doi:10.3390/diagnostics11030457)
Supplement: Supplementary file 1 [file diagnostics-11-00457-s001.pdf]

SUPPLEMENTARY TABLES

Table S1- Analysis of swab sampling at time T0 (before autopsy).

Cadaver	A.T. right*	A.T. left*	A.T. head*	A.T. feet*	F. S. 1*	F. S. 2*	A.R. wall 1*	A.R. wall 2*	A.R. wall 3*	A.R. wall 4*	A.R. floor*
**1**	Neg	Neg	Neg	Neg	Neg	Neg	Neg	Neg	Neg	Neg	Neg
**2**	Neg	Neg	Neg	Neg	Neg	Neg	Neg	Neg	Neg	Neg	Neg
**3**	Neg	Neg	Neg	Neg	Neg	Neg	Neg	Neg	Neg	Neg	Neg
**4**	Neg	Neg	Neg	Neg	Neg	Neg	Neg	Neg	Neg	Neg	Neg
**5**	Neg	Neg	Neg	Neg	Neg	Neg	Neg	Neg	Neg	Neg	Neg
**6**	Neg	Neg	Neg	Neg	Neg	Neg	Neg	Neg	Neg	Neg	Neg
**7**	Neg	Neg	Neg	Neg	Neg	Neg	Neg	Neg	Neg	Neg	Neg
**8**	Neg	Neg	Neg	Neg	Neg	Neg	Neg	Neg	Neg	Neg	Neg
**9**	Neg	Neg	Neg	Neg	Neg	Neg	Neg	Neg	Neg	Neg	Neg
**10**	Neg	Neg	Neg	Neg	Neg	Neg	Neg	Neg	Neg	Neg	Neg
**11**	Neg	Neg	Neg	Neg	Neg	Neg	Neg	Neg	Neg	Neg	Neg
**12**	Neg	Neg	Neg	Neg	Neg	Neg	Neg	Neg	Neg	Neg	Neg
**13**	Neg	Neg	Neg	Neg	Neg	Neg	Neg	Neg	Neg	Neg	Neg
**14**	Neg	Neg	Neg	Neg	Neg	Neg	Neg	Neg	Neg	Neg	Neg
**15**	Neg	Neg	Neg	Neg	Neg	Neg	Neg	Neg	Neg	Neg	Neg
**16**	Neg	Neg	Neg	Neg	Neg	Neg	Neg	Neg	Neg	Neg	Neg

Table S2- Analysis of swab sampling at time T1.

Cadaver	PMI (day)	Right bronchus	Left bronchus	A.T. right	A.T. left	A.T. head	A.T. feet	F. S. 1	F. S. 2	A.R. wall 1	A.R. wall 2	A.R. wall 3	A.R. wall 4	A.R. floor
**1**	54	Pos	Pos	Pos	Pos	Pos	Pos	Neg	Pos	Neg	Neg	Pos	Neg	Pos
**2**	58	Pos	Pos	Pos	Pos	Pos	Pos	Pos	Neg	Neg	Neg	Neg	Neg	Pos
**3**	54	Pos	Pos	Pos	Pos	Pos	Pos	Neg	Neg	Neg	Neg	Neg	Neg	Pos
**4**	61	Pos	Pos	Pos	Pos	Pos	Pos	Pos	Neg	Neg	Neg	Neg	Neg	Neg
**5**	64	Pos	Pos	Pos	Pos	Pos	Pos	Neg	Neg	Neg	Neg	Neg	Neg	Neg
**6**	50	Pos	Pos	Neg	Pos	Pos	Pos	Neg	Neg	Neg	Neg	Neg	Neg	Neg
**7**	78	Pos	Pos	Neg	Neg	Neg	Pos	Neg	Neg	Neg	Neg	Neg	Neg	Neg
**8**	1	Pos	Pos	Neg	Neg	Pos	Neg	Neg	Neg	Neg	Neg	Neg	Neg	Neg
**9**	5	Pos	Pos	Pos	Pos	Pos	Pos	Neg	Neg	Neg	Neg	Neg	Neg	Pos
**10**	1	Pos	Pos	Pos	Pos	Neg	Pos	Neg	Neg	Neg	Neg	Neg	Neg	Neg
**11**	1	Pos	Pos	Neg	Pos	Neg	Neg	Neg	Neg	Neg	Neg	Neg	Neg	Neg
**12**	1	Pos	Pos	Pos	Pos	Pos	Pos	Neg	Pos	Neg	Neg	Pos	Neg	Neg
**13**	1	Pos	Pos	Pos	Pos	Pos	Pos	Neg	Pos	Neg	Neg	Neg	Pos	Pos
**14**	1	Pos	Pos	Neg	Neg	Neg	Neg	Neg	Neg	Neg	Neg	Neg	Neg	Neg
**15**	1	Pos	Pos	Pos	Pos	Neg	Neg	Neg	Neg	Neg	Neg	Neg	Neg	Neg
**16**	1	Pos	Pos	Pos	Pos	Neg	Neg	Neg	Neg	Neg	Pos	Neg	Neg	Pos

PMI- Post Mortem Interval;
A.T. – Autopsy Table;
F.S. – Face Shield;
A.R.- Autopsy Room

Table S3- Analysis of swab sampling at time T2.

Cadaver	A.T. right*	A.T. left*	A.T. head*	A.T. feet*	F. S. 1*	F. S. 2*	A.R. wall 1*	A.R. wall 2*	A.R. wall 3*	A.R. wall 4*	A.R. floor*
**1**	Neg	Neg	Neg	Neg	Neg	Neg	Neg	Neg	Neg	Neg	Neg
**2**	Neg	Neg	Neg	Neg	Neg	Neg	Neg	Neg	Neg	Neg	Neg
**3**	Neg	Neg	Neg	Neg	Neg	Neg	Neg	Neg	Neg	Neg	Neg
**4**	Neg	Neg	Neg	Neg	Neg	Neg	Neg	Neg	Neg	Neg	Neg
**5**	Neg	Neg	Neg	Neg	Neg	Neg	Neg	Neg	Neg	Neg	Neg
**6**	Neg	Neg	Neg	Neg	Neg	Neg	Neg	Neg	Neg	Neg	Neg
**7**	Neg	Neg	Neg	Neg	Neg	Neg	Neg	Neg	Neg	Neg	Neg
**8**	Neg	Neg	Neg	Neg	Neg	Neg	Neg	Neg	Neg	Neg	Neg
**9**	Neg	Neg	Neg	Neg	Neg	Neg	Neg	Neg	Neg	Neg	Neg
**10**	Neg	Neg	Neg	Neg	Neg	Neg	Neg	Neg	Neg	Neg	Neg
**11**	Neg	Neg	Neg	Neg	Neg	Neg	Neg	Neg	Neg	Neg	Neg
**12**	Neg	Neg	Neg	Neg	Neg	Neg	Neg	Neg	Neg	Neg	Neg
**13**	Neg	Neg	Neg	Neg	Neg	Neg	Neg	Neg	Neg	Neg	Neg
**14**	Neg	Neg	Neg	Neg	Neg	Neg	Neg	Neg	Neg	Neg	Neg
**15**	Neg	Neg	Neg	Neg	Neg	Neg	Neg	Neg	Neg	Neg	Neg
**16**	Neg	Neg	Neg	Neg	Neg	Neg	Neg	Neg	Neg	Neg	Neg

*- Post Disinfection
